# Progressive structural bone changes and their relationship with treatment in patients with psoriatic arthritis: a longitudinal HR-pQCT study

**DOI:** 10.1186/s13075-019-2043-3

**Published:** 2019-12-04

**Authors:** Dongze Wu, James F. Griffith, Steven H. M. Lam, Priscilla C. H. Wong, Lin Shi, Edmund K. Li, Isaac T. Cheng, Tena K. Li, Vivian W. Hung, Ling Qin, Lai-Shan Tam

**Affiliations:** 1 Department of Medicine & Therapeutics, The Prince of Wales Hospital, The Chinese University of Hong Kong, Hong Kong, China; 2 Department of Imaging and Interventional Radiology, The Prince of Wales Hospital, The Chinese University of Hong Kong, Hong Kong, China; 3 Research Centre for Medical Image Computing, Department of Imaging and Interventional Radiology, The Prince of Wales Hospital, The Chinese University of Hong Kong, Hong Kong, China; 4 Bone Quality and Health Centre, Department of Orthopaedics and Traumatology, The Chinese University of Hong Kong, Hong Kong, China

**Keywords:** Psoriatic arthritis, Progression, Erosion, Enthesiophyte, Low disease activity

## Abstract

**Background:**

Although the short-term effects of tumor necrosis factor alpha (TNF-α) and interleukin-17A (IL-17A) inhibition on the structural changes in psoriatic arthritis (PsA) using high-resolution peripheral quantitative computed tomography (HR-pQCT) have been reported, no studies have investigated the long-term structural changes in PsA patients receiving routine care. We reported longitudinal changes of erosions and enthesiophytes using HR-pQCT and their relationship with treatments in PsA patients over a 5-year period.

**Methods:**

HR-pQCT examination at the second and third metacarpal heads (MCH2 and MCH3) was performed in 60 PsA patients at baseline and after 5 years. The size of each individual lesion was quantified. Erosion and enthesiophyte progression were defined as change exceeding the smallest detectable change (SDC).

**Results:**

A total of 108 bone erosions and 99 enthesiophytes were detected at baseline. Three new bone erosions but no new enthesiophytes were evident at 5 years. A significant increase in mean (±SD) erosion (0.58 ± 1.50 mm^3^, *P* < 0.001) and enthesiophyte (0.47 ± 0.76 mm^3^, *P* < 0.001) volume was observed. Erosion and enthesiophyte progression were found in 37/111 (33.3%) and 50/99 (50.5%) lesions, respectively. During this 5-year period, 26 (43%) out of the 60 patients achieved sustained Disease Activity index for PSoriatic Arthritis (DAPSA) low disease activity (LDA) (SDL group, defined as achieving DAPSA-LDA at both baseline and 5 years). Fourteen (23%) out of 60 patients received a TNF inhibitor throughout the 5-year period (TNFi group). Fewer erosions progressed (12/51 [23.5%] vs 25/60 [41.7%], *P* = 0.047) and the increased in enthesiophyte volume was significantly less (0.28 ± 0.67 vs 0.61 ± 0.80 mm^3^, *P* = 0.048) in the SDL group than in the non-SDL group. However, no significant difference between the TNFi and non-TNFi groups was detected in terms of the change in volume or progression of bone erosion and enthesiophyte.

**Conclusion:**

Damage accrual in terms of bone erosion and enthesiophyte was observed in PsA patients over a period of 5 years despite receiving routine clinical care. Nonetheless, sustained control of disease activity may be able to prevent these bony damages.

## Background

Psoriatic arthritis (PsA) is an inflammatory arthritis associated with psoriasis (PsO), involving not only the appendicular joints but also the entheses and the axial skeleton, resulting in bone damage [1, 2]. Chronic inflammation at these synovial and entheseal sites leads to erosions and enthesiophytes, respectively [1, 2]. By the time PsA patients first present to a rheumatologist, differences in bone erosion and enthesiophyte between PsA patients and healthy individuals are already evident [3–5]. There are age-related bone erosions and osteophytes in the hand joints of healthy individuals, suggesting that the threshold between “normal” and “pathological” is shifted with the increase of age [6]. However, whether there are progressive structural bone changes after the onset of PsA remains a topic of debate. Determining whether and how structural bone damages continue during the course of the PsA is important for understanding its pathophysiologic feature and has implications for the treatment.

Recent recommendations emphasized that remission or low disease activity (LDA) as assessed by Disease Activity index for PSoriatic Arthritis (DAPSA) and minimal disease activity (MDA) are the principal targets for the treatment of PsA [7, 8]. Tight control of PsA disease activity through a treat-to-target approach significantly improves joint outcomes for newly diagnosed patients [9]. Tumor necrosis factor (TNF) and interleukin-17A (IL-17A) are the key cytokines involved in both the inflammation and structural changes seen in PsA patients [10]. Using high-resolution peripheral quantitative computed tomography (HR-pQCT), short-term inhibition of erosion and enthesiophyte progression has been demonstrated in the metacarpophalangeal joints of PsA patients after IL-17 inhibition by secukinumab [11]. On the other hand, while erosions showed an arrest of progression in PsA patients treated with either methotrexate (MTX) or TNF inhibitor (TNFi), new bone formation progressed in both groups after 1 year [12].

We report herein the results of a longitudinal HR-pQCT study of PsA. We measured the change in bone erosion and enthesiophyte volumes over a period of 5 years using HR-pQCT. The aims of this study are (1) to characterize structural bone change in the course of PsA and (2) to determine how structural bone changes may relate to treatments in PsA patients.

## Methods

### Patients

Sixty consecutive PsA patients were included in this study. Baseline distal radial densitometric and microstructural features in 53 out of the 60 PsA patients have been published [13]. All PsA patients fulfilled the ClASsification criteria for Psoriatic ARthritis (CASPAR) and were rheumatoid factor negative. Patients with HR-pQCT-detected joint destruction were excluded [14]. All patients had a comprehensive clinical and HR-pQCT assessment at baseline and after 5 years.

All patients in this cohort received routine clinical care at the Prince of Wales Hospital. The choice of treatment, which included conventional synthetic disease-modifying anti-rheumatic drugs (csDMARDs) and biologic DMARDS (bDMARDs), was at the discretion of the patient and his or her treating rheumatologists. Ethics committee approval was obtained from the Ethics Committee of The Chinese University of Hong Kong-New Territories East Cluster Hospitals (CRE-2016.366). All patients provided written informed consent.

### Clinical assessment

Clinical and demographic parameters recorded include age, body weight, body height, and smoking habits. Clinical assessment included the number of swollen, tender, and deformed joints, Maastricht Ankylosing Spondylitis Enthesitis Score (MASES), and the presence of dactylitis. Disease activity was assessed using the Disease Activity index for PsA (DAPSA) [15], Psoriasis Area and Severity Index (PASI), erythrocyte sedimentation rate (ESR), and C-reactive protein (CRP). Physical function was assessed using the Health Assessment Questionnaire (HAQ) disability index. Use of non-steroidal anti-inflammatory drugs (NSAIDs), csDMARDs, and bDMARDs over the 5-year period was retrieved from the electronic management system.

Remission was defined as very low disease activity (VLDA) or DAPSA ≤4. LDA was defined as MDA or DAPSA ≤14. Sustained disease control was defined as achieving these targets at baseline and 5 years.

### High-resolution peripheral quantitative CT

All patients underwent HR-pQCT examination (XtremeCT scanner, SCANCO Medical AG, Brüttisellen, Switzerland) at a nominal isotropic voxel size of 82 × 82 × 82 μm of the metacarpophalangeal (MCP) joint of the non-dominant forearm at baseline and 5 years. The non-dominant forearm was chosen to avoid the effects of mechanical stress, similar to other studies [16]. Assessment was confined to the second and third metacarpal head (MCH) regions to optimize time efficiency and minimize motion artifact. The second and third MCHs are the frequently affected MCHs in PsA when assessed by HR-pQCT. Erosive lesions were more commonly found at the MCP2 (82.1%) and MCP3 (85.7%) joints than the MCP4 joint (57.0%) [17]. Similarly, osteophytes were more common at the MCP2 (82%) and MCP3 (86%) joints than at the MCP4 joint (50%) [17].

The patients’ forearm was immobilized in a carbon fiber cast fixed within the scanner gantry. A dorsopalmar projection image was obtained to define the tomographic scan region. The scan region started at the distal end of the MCH and spanned proximally 9.02 mm (110 slices) at baseline, extended between 80 slices distal to the edge of the MCH and 242 slices proximal to it at 5 years (322 slices).

### Co-localization of VOI: baseline-indexed image registration and slice matching

A fully automated baseline-indexed 3D image registration and re-slicing (slice matching) [18] based on ITK-SNAP [19] were performed to acquire precisely matched baseline and follow-up volumes of interest (VOIs) at the second and third metacarpal heads (MCH2 and MCH3) (Additional file 1: Figure S1). Follow-up scans were coarsely aligned based on image center and principal axes and registered onto the baseline images using rigid transformation and mutual information image similarity metrics. Then, these scans were re-sliced into the same space of the baseline image using linear interpolation to account for translation and rotation.

Slice-matched co-localized regions of interest (ROIs) were specified on the baseline and follow-up HR-pQCT examinations to assess longitudinal changes in erosions and enthesiophytes. The ROI criteria applied were to (1) include the metacarpal bone only, (2) exclude regions without bone, and (3) exclude the proximal or distal three slices to obviate boundary effect, if necessary.

### Image analysis

The MCP joints were evaluated for the following criteria: number, size, and volume of erosions and enthesiophytes on the palmar, ulnar, dorsal, and radial quadrants of the MCH. Enthesiophytes were defined as new bone formation arising from the periosteal bone cortex at the insertion sites of the capsule, ligament, or tendons or at the location of functional enthesis [5, 20]. Erosions were defined as a clear break in the outer cortical margin evident on at least two consecutive slices and in two orthogonal planes [5, 17]. Two compartments were imaged corresponding to MCH2 and MCH3 for each patient. A semi-automated method based on ITK-SNAP [21] was used to calculate erosion and enthesiophyte volume on co-localized baseline and follow-up VOIs [4].

### Smallest detectable change

To determine the smallest detectable change (SDC), 25 enthesiophytes and 30 erosions were randomly chosen and scored twice. SDC was then calculated for enthesiophyte and erosion volume using the formula SDC = ±1.96 × SD_Δ(CHANGE-SCORES)_ / (√2 × √*k*), where _Δ(CHANGE-SCORES)_ is the standard deviation of change in scores and *k* the number of readings [22].

### Definition of progression of bone erosion or enthesiophyte

Erosion or enthesiophyte progression was defined as either (i) an increase in erosion or enthesiophyte volume exceeding SDC (SDC for erosion: 0.5 mm^3^, enthesiophyte: 0.3 mm^3^) or (ii) new development of an erosion or enthesiophyte. Erosion or enthesiophyte regression was defined as a decrease in erosion or enthesiophyte volume exceeding SDC. Individual erosions or enthesiophytes which did not fulfill the progression or regression criteria were classified as stable.

### Outcomes

The purpose of this cohort study was (1) to determine the longitudinal change in erosion and enthesiophyte volume over a period of 5 years and (2) to evaluate the effect of (i) achieving sustained DAPSA-LDA (SDL) and (ii) long-term anti-TNF therapy on the progression of erosions and enthesiophytes.

### Statistical analysis

After testing for Gaussian distribution, paired *t* test or Wilcoxon signed-rank test was applied to compare the structural bone changes at baseline and 5 years as appropriate. Comparisons between the two groups were performed using the chi-square test for categorical variables. Generalized mixed linear models were used to adjust for confounding factors including gender, age, body mass index (BMI), disease duration, disease activity (DAPSA) at baseline, and treatment over the 5-year period. Data were analyzed using the Statistical Package for the Social Sciences software for statistics (IBM SPSS V.22.0, IBM Corporation, Armonk, NY, USA), and a *P* value of less than 0.05 was considered statistically significant.

## Results

### Demographic and clinical features

At baseline, the mean age of the cohort was 51.9 ± 8.9 years, 27 (45.0%) were female, and 13 (21.7%) were ever-smokers (Table 1). The mean duration of psoriasis and PsA before study entry was 18.7 ± 16.7 years and 14.3 ± 6.9 years. Twenty-nine patients (48.3%) had radiologic evidence of juxta-articular new bone formation. Most patients had mild to moderate disease activity. Three (5.0%) patients received corticosteroids, 44 (73.3%) were on csDMARDs, and 16 (26.7%) received bDMARDs.

**Table 1:** Changes in demographic and clinical characteristics over 5-year

	Baseline (*n* = 60)	5 years (*n* = 60)	Change (*n* = 60)	*P* value
Demographic characteristics				
Female, *N* (%)	27 (45.0)			
Smokers, *N* (%)	13 (21.7)			
Age, year	51.9 ± 8.9			
BMI	25.4 ± 4.3	25.8 ± 4.5	0.46 ± 1.9	0.060
Height (cm)	163.6 ± 8.5	163.0 ± 8.4	−0.57 ± 1.3	*0.002*
Weight (kg)	68.2 ± 14.3	68.8 ± 14.0	0.62 ± 5.1	0.345
Disease-specific characteristics				
Duration of psoriasis (year)	18.7 ± 16.7			
Duration of PsA (year)	14.3 ± 6.9			
Juxta-articular new bone formation	29 (48.3)			
Polyarticular	21 (35.0)			
Oligoarticular	17 (28.3)			
Presence of enthesitis	13 (21.7)			
Presence of dactylitis	8 (13.3)			
Tender joint at MCP2	8 (13.3)			
Tender joint at MCP3	4 (6.6)			
Swollen joint at MCP2	3 (5.0)			
Swollen joint at MCP3	2 (3.3)			
CRP (mg/L)	4.4 ± 4.9	4.0 ± 5.2	−0.4 ± 5.7	0.629
ESR (mm/h)	19.0 ± 15.9	27.5 ± 17.4	8.7 ± 13.3	*0.000*
DAPSA	11.4 ± 9.3	9.7 ± 5.9	−1.7 ± 9.0	0.150
PASI	6.3 ± 8.6	4.7 ± 6.4	−1.6 ± 7.2	0.088
HAQ	0.3 ± 0.4	0.4 ± 0.5	0.1 ± 0.4	0.067
MASES	0.9 ± 2.4	0.5 ± 1.4	−0.5 ± 1.8	*0.035*
VAS pain (mm)	31.1 ± 26.1	31.1 ± 24.5	0 ± 26.4	1.000
VAS PhyGA (mm)	23.8 ± 18.9	18.5 ± 18.2	−5.4 ± 21.8	0.060
VAS PatGA (mm)	41.0 ± 28.3	37.0 ± 22.9	−4.0 ± 26.0	0.243
Tender joint count	2.9 ± 5.7	1.8 ± 2.8	−1.2 ± 5.4	0.103
Swollen joint count	0.8 ± 1.5	0.7 ± 1.6	−0.1 ± 1.7	0.597
Deformed joint count	3.8 ± 6.4	7.0 ± 7.4	3.2 ± 5.5	*0.000*
Current/ever treatment				
NSAIDs, *N* (%)	25 (41.7)	28 (46.7)		0.080
Corticosteroids, *N* (%)	3 (5.0)	4 (6.7)		1.000
csDMARDs, *N* (%)	44 (73.3)	49 (81.7)		0.274
bDMARDs, *N* (%)	16 (26.7)	23 (35.3)		0.172
Any DMARDs, *N* (%)	46 (76.7)	53 (88.3)		0.093
csDMARDs + bDMARDs, *N* (%)	16 (26.7)	19 (31.7)		0.547
Clinical response				
VLDA, *N* (%)	4 (6.7)	8 (13.3)		0.083
MDA, *N* (%)	15 (25.0)	22 (36.7)		0.166
REM (DAPSA ≤4), *N* (%)	14 (23.3)	13 (21.7)		*0.044*
LDA (DAPSA ≤14), *N* (%)	26 (43.3)	29 (48.3)		
MDA (DAPSA ≤28), *N* (%)	17 (28.3)	18 (30.0)		
HDA (DAPSA >28), *N* (%)	3 (5.0)	0 (0)		

Results are mean ± SD or number (percentage) unless otherwise indicated. Significant results are highlighted in italics

*BMI* body mass index, *MCP* metacarpophalangeal joint, *CRP* C-reactive protein, *ESR* erythrocyte sedimentation rate, *DAPSA* Disease Activity in PSoriatic Arthritis, *PASI* Psoriasis Area and Severity Index, *HAQ* Health Assessment Questionnaire, *VAS* Visual Analogue Scale, *PhyGA* Physician Global Assessment, *PatGA* Patient Global Assessment, *NSAIDs* non-steroidal anti-inflammatory drugs, *csDMARDs* conventional synthetic disease-modifying anti-rheumatic drugs, *bDMARDs* biologic disease-modifying anti-rheumatic drugs, *VLDA* very low disease activity, *MDA* minimal disease activity, *REM* remission, *LDA* low disease activity, *MDA* moderate disease activity, *HDA* high disease activity

Compared with baseline, disease activity at 5 years remained relatively stable as only seven patients were started on bDMARDs. Fourteen (23%) patients received TNFi with (*n* = 6) or without (*n* = 8) concomitant methotrexate (MTX) throughout the 5-year period. Seven patients received TNFi less than 5 years, two patients received ustekinumab and secukinumab for less than 2 years, including one patient who switched from ustekinumab to secukinumab. No patient received bone-active drugs including denosumab.

Twenty-six (43%) patients achieved sustained DAPSA-LDA ([SDL], defined as achieving DAPSA-LDA at baseline and at 5 years) while 9 (15%) patients achieved sustained minimal disease activity ([sMDA], defined as achieving MDA at baseline and at 5 years).

### Change in bone erosion and enthesiophyte after 5 years

There were 108 erosions and 99 enthesiophytes at baseline. After 5 years, three new erosions but no new enthesiophytes were evident (Table 2). There was a significant increase in the volume of individual erosions (0.6 ± 1.5 mm^3^, *P* < 0.001) (Additional file 1: Figure S2A) and enthesiophytes (0.5 ± 0.8 mm^3^, *P* < 0.001) (Additional file 1: Figure S2B).

**Table 2:** Number and volume of bone erosion and enthesiophyte

	Baseline (*n* = 60)	5 years (*n* = 60)	Change (*n* = 60)	*P* value
Number				
Erosions total, *N*	108	111	3	
Metacarpal head 2, *N* (%)	49 (45.4)	50 (45.1)	1	
Metacarpal head 3, *N* (%)	59 (54.6)	61 (54.9)	2	
Enthesiophytes total, *N*	99	99	0	
Metacarpal head 2, *N* (%)	58 (58.6)	58 (58.6)	0	
Metacarpal head 3, *N* (%)	41 (41.4)	41 (41.4)	0	
Volume of each individual lesion				
Erosions				
Metacarpal head 2 (mm^3^)	3.6 ± 4.6	4.0 ± 4.8	0.4 ± 1.5	*0.050*
Metacarpal head 3 (mm^3^)	4.8 ± 3.7	5.5 ± 4.3	0.7 ± 1.5	*0.001*
Metacarpal heads 2 and 3 (mm^3^)	4.2 ± 4.2	4.8 ± 4.6	0.6 ± 1.5	*<0.001*
Enthesiophytes				
Metacarpal head 2 (mm^3^)	3.3 ± 2.2	3.7 ± 2.4	0.4 ± 0.7	*<0.001*
Metacarpal head 3 (mm^3^)	3.5 ± 2.5	4.0 ± 2.8	0.5 ± 0.9	*<0.001*
Metacarpal heads 2 and 3 (mm^3^)	3.4 ± 2.3	3.9 ± 2.6	0.5 ± 0.8	*<0.001*

Results are mean ± SD or number (percentage). Significant results are highlighted in italics

**Fig. 1:**
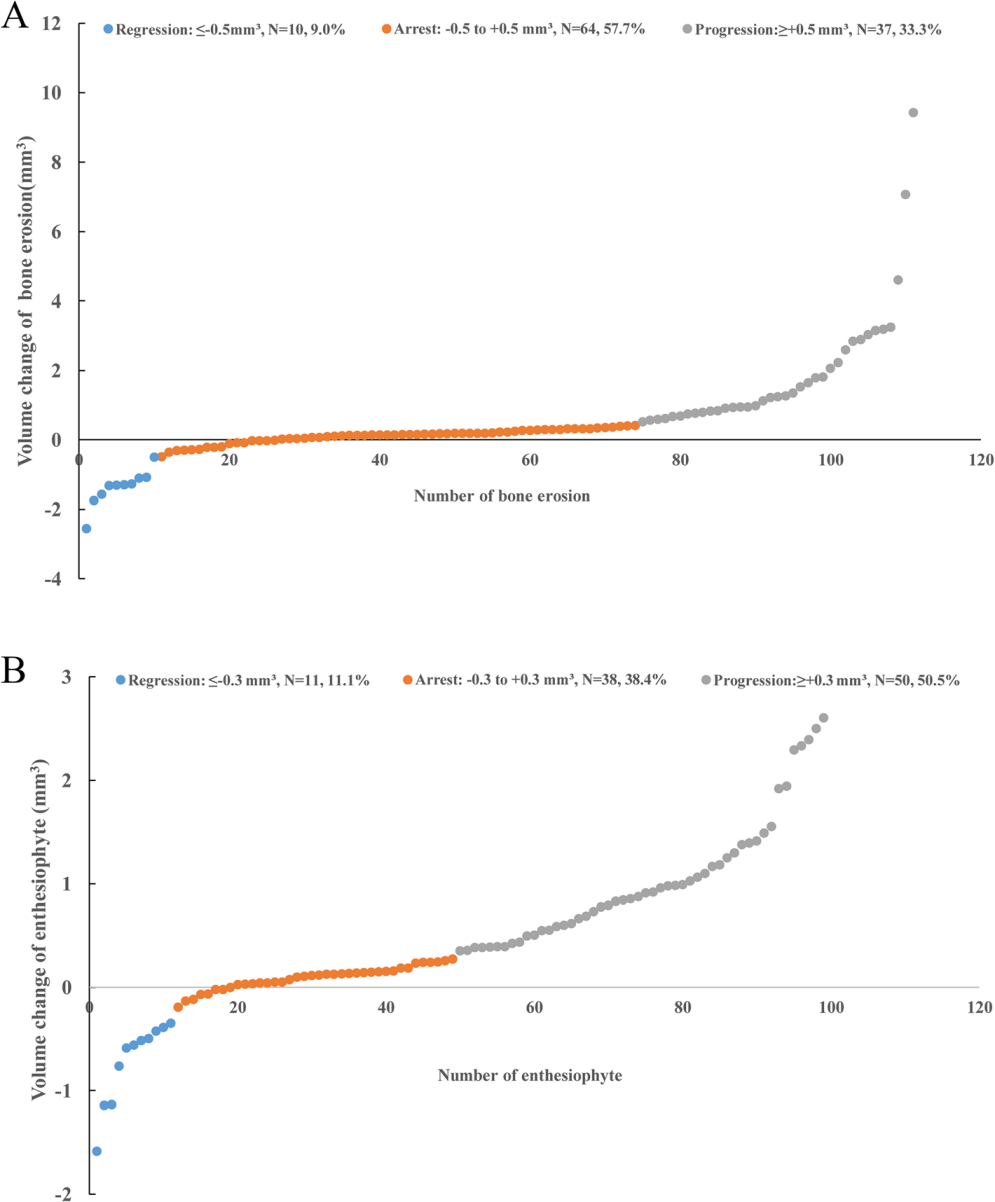
Cumulative probability plot of each bone erosion (**a**) and enthesiophyte (**b**). Each dot represents one bone erosion or enthesiophyte. The *x*-axis indicates the number of individual bone erosion or enthesiophyte; the *y*-axis indicates the volume change for individual erosion or enthesiophyte from baseline to 5 years. Blue dots indicate regression, orange dots indicate stable, and gray dots indicate progression in bone erosion or enthesiophyte

Probability plots showed a clear shift to progression of erosion volume (>0.5 mm^3^ increase in volume: *N* = 37/111 (33.3%) (Fig. 1a). The mean ± SD increase in erosion volume in this subgroup was 1.9 ± 1.8 mm^3^. Erosion volume was stable in 64/111 (57.7%), with a change in size of 0.1 ± 0.2 mm^3^. Only 10/111 (9.0%) erosions showed evidence of regression (>0.5 mm^3^ decrease in volume) with a change in size of −1.4 ± 0.5 mm^3^.

Similarly, probability plots showed a clear shift to progression of enthesiophyte volume (>0.3 mm^3^ increase in volume: *N* = 50/99 [50.5%]) (Fig. 1b). The mean ± SD increase in volume of this subgroup was 1.0 ± 0.6 mm^3^. Enthesiophyte volume was stable in 38/99 (38.4%), with a change in size of 0.1 ± 0.1 mm^3^. Only a few enthesiophytes showed evidence of regression (>0.3 mm^3^ decrease in size: *N* = 11/99 [11.1%]), with a change in size of −0.7 ± 0.4 mm^3^.

Erosions or enthesiophytes that showed progression have a significantly larger baseline volume compared to those lesions which showed no progression (either stable or regression) (Additional file 1: Figure S3).

### Effects of achieving sustained DAPSA-LDA

The change in erosion volume was similar in patients who achieved sustained DAPSA-LDA (SDL group, *n* = 26) and those who did not achieve sustained DAPDA-LDA (non-SDL group, *n* = 34) (Table 3, Additional file 1: Figure S4-A). However, a significantly lower proportion of erosions progressed in the SDL group compared with the non-SDL group (12/51 [23.5%] vs 25/60 [41.7%], *P* = 0.047) (Table 3).

**Table 3:** Number of lesions with progression and the change in volume of the lesions over 5 years

	SDL group (*n* = 26)	Non-SDL group (*n* = 34)	*P* value	TNFi group (*n* = 14)	Non-TNFi group (*n* = 46)	*P* value
Erosions, *N* (%)						
Progression	12/51 (23.5)	25/60 (41.7)	*0.043*	8/26 (30.8)	29/85 (34.1)	0.751
Non-progression	39/51 (76.5)	35/60 (58.3)		18/26 (69.2)	56/85 (65.9)	
Enthesiophytes, *N* (%)						
Progression	17/40 (42.5)	33/59 (55.9)	0.190	15/29 (51.7)	36/70 (51.4)	0.979
Non-progression	23/40 (57.5)	26/59 (44.1)		14/29 (48.3)	34/70 (48.6)	
Mean change of volume						
Erosions (mm^3^)	0.5 ± 1.6	0.7 ± 1.4	0.100	0.3 ± 1.2	0.7 ± 1.6	0.638
Enthesiophytes (mm^3^)	0.3 ± 0.7	0.6 ± 0.8	*0.048*	0.4 ± 0.7	0.5 ± 0.8	0.620

Results are mean ± SD or number (percentage). Significant results are highlighted in italics

*SDL group* achieved sustained DAPSA-LDA, *non-SDL group* did not achieve sustained DAPSA-LDA, *TNFi group* patients who received TNF inhibitor throughout 5 years, *non-TNFi group* patients who did not receive TNF inhibitor throughout 5 years

Similarly, increase in enthesiophyte volume in the SDL group was significantly lower than that in the non-SDL group (mean ± SD change: 0.3 ± 0.7 vs 0.6 ± 0.8, *P* = 0.048) (Table 3, Additional file 1: Figure S4-B). Although a numerically lower proportion of enthesiophytes progressed in the SDL group compared to the non-SDL group (17/40 [42.5%] vs 33/59 [55.9%], *P* = 0.153), the difference did not reach statistical significance (Table 3). Using the generalized mixed linear model, only sustained DAPSA-LDA is negatively associated with the change in total enthesiophyte volume after adjusting for the use of TNFi and other baseline parameters (Additional file 1: Table S1).

### Effects of long-term anti-TNF therapy

The change in size of erosions and enthesiophytes was similar in the long-term TNFi (*n* = 14) and non-TNFi (*n* = 46) patient groups (Table 3, Additional file 1: Figure S5). A similar proportion of erosions and enthesiophytes progressed in the TNFi and non-TNFi groups (Table 3). These results were unchanged after excluding the seven patients who received <5 years of TNFi and other bDMARDs in the non-TNFi group (results not shown).

## Discussion

To the best of our knowledge, no previous longitudinal HR-pQCT studies have reported the long-term change in bone erosion and enthesiophyte in PsA patients receiving routine care. We observed progressive increase in bone erosion and enthesiophyte volume in PsA patients, which might be prevented by sustained control of disease activity. Unfortunately, we did not have the longitudinal data of a healthy control group for comparison. One cross-sectional study revealed a 4% increase in erosion and osteophyte count, as well as erosion volume per year in the healthy subjects [6], which was much greater than the rate of change observed in the present study. Thus, how the longitudinal structural bone changes in PsA differ from the usual age-related trajectory in heathy subjects [6] needs further investigation.

Radiography is currently the main modality used to monitor structural damage in peripheral spondyloarthritis (SpA) including PsA [23], though commonly used scoring systems do not include bony proliferation as a criterion [24–27]. Although 91 to 98% of PsA patients across all trial groups met the radiographic criteria for non-progression in recent studies [28], our data demonstrated that structural bone damage accrual continued despite conventional treatment in terms of bone erosion and enthesiophyte. More effective treatments and strategies will need to be developed to prevent progression of bony damage.

More importantly, it provides preliminary evidence that long-term control of disease activity (achieving sustained DASPA-LDA) using various combinations of NSAIDs, csDMARDs, and bDMARDs may be useful in preventing the progression of enthesiophytes and bone erosions. Our findings concurred with previous studies reporting significantly greater radiographic progression in subjects with a moderate or poor outcome utilizing different composite disease activity measures including DAPSA over a period of 6 months [29]. Similar to other studies in newly diagnosed PsA patients [30], the proportion of PsA patients who achieved DAPSA-LDA was much higher than MDA after 5-year routine care. Higher DAPSA scores at 6 months were significantly and independently associated with probability of radiographic structural progression over a period of 1 year after adjusting for baseline radiographic score and treatment received [15]. Our findings add significant new information about the importance of sustained control of PsA disease activity, not only for improvement in pain and function but also for prevention of anabolic and catabolic structural joint damage.

Our data failed to detect a significant difference between patients with and without TNFi treatment throughout 5 years in terms of bone erosion and enthesiophyte, which confirmed previous findings that TNFi cannot inhibit enthesiophyte formation in PsA [12]. Inflammatory cytokines including TNF-α, IL-17, IL-23, and IL-22 can increase osteogenic differentiation [31]. Nonetheless, the overall impact of these cytokines on pathologic bone formation is not well understood [31]. For example, IL-22 enhances while a combination of TNF and interferon-ɣ (IFN-ɣ) with IL-22 suppresses osteogenic differentiation in human mesenchymal stem cells [32]. One can appreciate that IL-17 and TNF-α can inhibit or promote bone formation in a context-dependent manner. Therefore, it is not surprising that TNFi does not inhibit enthesiophyte formation in PsA as seen in the current study. Indeed, prospective studies have shown progression of spinal syndesmophyte formation over 2 years in patients with ankylosing spondylitis (AS) despite ongoing TNFi therapy [33, 34]. That said, recent retrospective analyses have suggested that long-term TNFi therapy can retard radiographic progression [35–37], while a prospective cohort of axial SpA patients also demonstrated a reduced likelihood of new syndesmophyte formation with TNFi use [38]. Further studies will elucidate whether longer term use (>5 years) or earlier use (disease duration <10 years) of TNFi will be effective in preventing enthesiophyte formation in PsA, similar to what has been shown in the axial skeleton.

Receptor activator of nuclear factor-kappa Β (RANK) ligand (RANKL), in the presence of macrophage colony-stimulating factor, is a major factor involved in osteoclastogenesis. TNF-α can amplify osteoclastogenesis in the presence of RANKL as well as having a direct effect on osteoclast formation when RANKL is not present [31]. Given the known capacity of TNF-α to promote osteoclastogenesis [39], one would expect suppression of this cytokine may reduce structural bone damage. A previous study reported no change in the width of erosion in both MTX- and TNFi-treated groups after 1 year, while the depth of the erosion even decreased slightly in the latter group [12]. In the current study, the increase in erosion size was numerically less in the TNFi group compared with the non-TNFi group, although statistically insignificant, probably due to the small sample size and long disease duration. Structural bone changes are generally more active in early disease [40]; therefore, the effects of anti-TNF in preventing erosion progression may be less obvious in our group of patients with long-standing disease compared to the cohort with shorter disease duration [12]. Retrospective studies have demonstrated that patients with erosive PsA receiving TNFi had a better radiographic outcome compared to those treated with MTX [41]. A recent randomized trial showed that etanercept-containing arms showed less radiographic progression compared with MTX monotherapy at week 48 [42]. Future studies with a larger sample size will be required to confirm whether TNFi is superior to MTX in preventing long-term erosion progression in PsA using HR-pQCT. Six-month treatment of secukinumab prevented erosion and enthesiophyte progression on HR-pQCT and reduced disease activity evident on magnetic resonance imaging in PsA patients [11]. We were not able to assess the effects of secukinumab as only two patients received this treatment, and the majority of our patients were treated with csDMARDs and NSAIDs.

This is the first long-term follow-up study in a PsA cohort to study the progression of catabolic and anabolic bone changes using HR-pQCT. We perform baseline-indexed image registration [43] and slice matching to acquire precisely matched baseline and follow-up VOI [44] to ensure reliability. In addition, we assessed the change in enthesiophyte volume instead of measuring maximal height [12] or using semi-quantitative grading methods [11, 17], which should provide a more objective assessment.

One limitation of this study is that validated disease activity assessments, e.g., DAPSA, were not routinely performed except at baseline and 5 years. Future longitudinal studies with serial disease activity assessments are needed to provide further information on the role of cumulative inflammatory burden and the development of bone damage in PsA. We also did not investigate minimal clinical meaningful volume change of bone erosion or enthesiophyte, especially for hand function. We acknowledged that the number of TNF-treated patients might not be sufficient to reach a solid conclusion. Thus, a well-designed long-term cohort study with a large sample size should be used to further address this question. Moreover, we did not repeat x-rays of all the hands and feet at 5 years, which might be useful to correlate radiologic new bone formation with HR-pQCT findings in future studies. Last but not least, we did not detect any differences in the risk of bone damage progression in patients with and without tender or swollen joints at baseline. Imaging modalities including magnetic resonance imaging (MRI) or ultrasonography would have been useful to objectively document and quantify the burden of inflammation. A multi-modality imaging study would be important to address whether synovitis confirmed by MRI or ultrasound at baseline may predict the development of bone erosion and enthesiophyte on HR-pQCT in the future.

## Conclusions

Damage accrual in terms of bone erosion and enthesiophyte was observed in PsA patients over a period of 5 years despite receiving routine clinical care. Nonetheless, sustained control of disease activity may be able to prevent these bony damages. Future larger long-term HR-pQCT studies are needed to validate these initial observations.

## Supplementary information

**Additional file 1 : Figure S1.** Representative image of baseline indexed image registration and slice matching**. Figure S2**. Examples of bone erosion and enthesiophyte in psoriatic arthritis (PsA) patients at baseline and 5-year follow-up**. Figure S3.** Baseline volume and dynamics of bone erosion and enthesiophyte**. Figure S4.** Mean within-subject volume changes in bone erosion and enthesiophyte between patients who achieved and did not achieve sustained DAPSA-LDA. **Figure S5.** Mean within-subject volume changes in bone erosion and enthesiophyte between patients with and without TNFi throughout 5-year**. Table S1.** Generalized mixed linear model with change in bone erosion and enthesiophyte volume as dependent variable.

## Data Availability

The datasets used and/or analyzed during the present study are available from the corresponding author on reasonable request.

## Abbreviations

bDMARDs: Biologic disease-modifying anti-rheumatic drugs
BMI: Body mass index
CASPAR: ClASsification criteria for Psoriatic ARthritis
CRP: C-reactive protein
csDMARDs: Conventional synthetic disease-modifying anti-rheumatic drugs
DAPSA: Disease Activity index for PsA
ESR: Erythrocyte sedimentation rate
HAQ: Health Assessment Questionnaire
HR-pQCT: High-resolution peripheral quantitative CT
IL: Interleukin
LDA: Low disease activity
MASES: Maastricht Ankylosing Spondylitis Enthesitis Score
MCH: Metacarpal heads
MDA: Minimal disease activity
NSAIDs: Non-steroidal anti-inflammatory drugs
PASI: Psoriasis Area and Severity Index
PsA: Psoriatic arthritis
PsO: Psoriasis
SDC: Smallest detectable change
TNF: Tumor necrosis factor
VLDA: Very low disease activity

## References

[1] Gladman DD, Antoni C, Mease P, Clegg DO, Nash P (2005). Psoriatic arthritis: epidemiology, clinical features, course, and outcome. Ann Rheum Dis.

[2] Ritchlin CT, Colbert RA, Gladman DD (2017). Psoriatic arthritis. New Engl J Med.

[3] Simon D, Kleyer A, Faustini F, Englbrecht M, Haschka J, Berlin A, Kraus S, Hueber AJ, Kocijan R, Sticherling M (2018). Simultaneous quantification of bone erosions and enthesiophytes in the joints of patients with psoriasis or psoriatic arthritis - effects of age and disease duration. Arthritis Res Ther.

[4] Wu D, Griffith JF, Lam SHM, Yue J, Wong P, Shi L, Li E, Cheng IT, Li TK, Zhu TY, Hung VW, Qin L, Tam LS. Structural and Microstructural. Intraarticular Bone Changes at the Metacarpal Heads in Patients with Psoriatic Arthritis Compared to Controls: A HR-pQCT Study [abstract]. Arthritis Rheumatol. 2018;70(suppl):949–51.

[5] Simon D, Faustini F, Kleyer A, Haschka J, Englbrecht M, Kraus S, Hueber AJ, Kocijan R, Sticherling M, Schett G (2016). Analysis of periarticular bone changes in patients with cutaneous psoriasis without associated psoriatic arthritis. Ann Rheum Dis.

[6] Berlin A, Simon D, Tascilar K, Figueiredo C, Bayat S, Finzel S, Klaus E, Rech J, Hueber AJ, Kleyer A (2019). The ageing joint-standard age- and sex-related values of bone erosions and osteophytes in the hand joints of healthy individuals. Osteoarthritis Cartilage.

[7] Singh JA, Guyatt G, Ogdie A, Gladman DD, Deal C, Deodhar A, Dubreuil M, Dunham J, Husni ME, Kenny S (2019). Special Article: 2018 American College of Rheumatology/National Psoriasis Foundation guideline for the treatment of psoriatic arthritis. Arthritis Rheumatol.

[8] Smolen JS, Schols M, Braun J, Dougados M, FitzGerald O, Gladman DD, Kavanaugh A, Landewe R, Mease P, Sieper J (2018). Treating axial spondyloarthritis and peripheral spondyloarthritis, especially psoriatic arthritis, to target: 2017 update of recommendations by an international task force. Ann Rheum Dis.

[9] Coates LC, Moverley AR, McParland L, Brown S, Navarro-Coy N, O'Dwyer JL, Meads DM, Emery P, Conaghan PG, Helliwell PS (2015). Effect of tight control of inflammation in early psoriatic arthritis (TICOPA): a UK multicentre, open-label, randomised controlled trial. Lancet..

[10] Gravallese EM, Schett G (2018). Effects of the IL-23–IL-17 pathway on bone in spondyloarthritis. Nat Rev Rheumatol.

[11] Kampylafka E, d'Oliveira I, Linz C, Lerchen V, Stemmler F, Simon D, Englbrecht M, Sticherling M, Rech J, Kleyer A (2018). Resolution of synovitis and arrest of catabolic and anabolic bone changes in patients with psoriatic arthritis by IL-17A blockade with secukinumab: results from the prospective PSARTROS study. Arthritis Res Ther.

[12] Finzel S, Kraus S, Schmidt S, Hueber A, Rech J, Engelke K, Englbrecht M, Schett G (2013). Bone anabolic changes progress in psoriatic arthritis patients despite treatment with methotrexate or tumour necrosis factor inhibitors. Ann Rheum Dis.

[13] Zhu TY, Griffith JF, Qin L, Hung VW, Fong TN, Au SK, Kwok AW, Leung PC, Li EK, Tam LS (2015). Density, structure, and strength of the distal radius in patients with psoriatic arthritis: the role of inflammation and cardiovascular risk factors. Osteoporos Int.

[14] Stach CM, Bauerle M, Englbrecht M, Kronke G, Engelke K, Manger B, Schett G (2010). Periarticular bone structure in rheumatoid arthritis patients and healthy individuals assessed by high-resolution computed tomography. Arthritis Rheum.

[15] Aletaha D, Alasti F, Smolen JS (2017). Disease activity states of the DAPSA, a psoriatic arthritis specific instrument, are valid against functional status and structural progression. Ann Rheum Dis.

[16] Sornay-Rendu E, Boutroy S, Munoz F, Delmas PD (2007). Alterations of cortical and trabecular architecture are associated with fractures in postmenopausal women, partially independent of decreased BMD measured by DXA: the OFELY study. J Bone Miner Res.

[17] Finzel S, Englbrecht M, Engelke K, Stach C, Schett G (2011). A comparative study of periarticular bone lesions in rheumatoid arthritis and psoriatic arthritis. Ann Rheum Dis.

[18] Geusens P, Chapurlat R, Schett G, Ghasem-Zadeh A, Seeman E, de Jong J, van den Bergh J (2014). High-resolution in vivo imaging of bone and joints: a window to microarchitecture. Nat Rev Rheumatol.

[19] Yushkevich PA, Gerig G (2017). ITK-SNAP: an intractive medical image segmentation tool to meet the need for expert-guided segmentation of complex medical images. IEEE Pulse.

[20] Faustini F, Simon D, Oliveira I, Kleyer A, Haschka J, Englbrecht M, Cavalcante AR, Kraus S, Tabosa TP, Figueiredo C (2016). Subclinical joint inflammation in patients with psoriasis without concomitant psoriatic arthritis: a cross-sectional and longitudinal analysis. Ann Rheum Dis.

[21] Yushkevich PA, Yang G, Gerig G (2016). ITK-SNAP: an interactive tool for semi-automatic segmentation of multi-modality biomedical images. Conf Proc IEEE Eng Med Biol Soc.

[22] Bruynesteyn K, Boers M, Kostense P, van der Linden S, van der Heijde D (2005). Deciding on progression of joint damage in paired films of individual patients: smallest detectable difference or change. Ann Rheum Dis.

[23] Mandl P, Navarro-Compan V, Terslev L, Aegerter P, van der Heijde D, D'Agostino MA, Baraliakos X, Pedersen SJ, Jurik AG, Naredo E (2015). EULAR recommendations for the use of imaging in the diagnosis and management of spondyloarthritis in clinical practice. Ann Rheum Dis.

[24] Marchesoni A, Caporali R, Lubrano E (2019). Clinical implications of peripheral new bone formation in psoriatic arthritis: a literature-based review. Clin Exp Rheumatol.

[25] Wassenberg S (2015). Radiographic scoring methods in psoriatic arthritis. Clin Exp Rheumatol.

[26] van der Heijde D, Sharp J, Wassenberg S, Gladman DD (2005). Psoriatic arthritis imaging: a review of scoring methods. Ann Rheum Dis.

[27] Salaffi F, Carotti M, Beci G, Di Carlo M, Giovagnoni A. Radiographic scoring methods in rheumatoid arthritis and psoriatic arthritis. Radiol Med. 2019;124(11):1071–86. 10.1007/s11547-019-01001-3 30739290

[28] Mease P, Hall S, FitzGerald O, van der Heijde D, Merola JF, Avila-Zapata F, Cieslak D, Graham D, Wang C, Menon S (2017). Tofacitinib or adalimumab versus placebo for psoriatic arthritis. New Engl J Med.

[29] Helliwell PS, Kavanaugh A (2018). Radiographic progression in psoriatic arthritis achieving a good response to treatment: data using newer composite indices of disease activity. Arthritis Care Res.

[30] Wervers K, Vis M, Tchetveriko I, Gerards AH, Kok MR, Appels CWY, van der Graaff WL, van Groenendael J, Korswagen LA, Veris-van Dieren JJ (2018). Burden of psoriatic arthritis according to different definitions of disease activity: comparing minimal disease activity and the disease activity index for psoriatic arthritis. Arthritis Care Res.

[31] Paine A, Ritchlin C (2018). Altered bone remodeling in psoriatic disease: new insights and future directions. Calcif Tissue Int.

[32] El-Zayadi AA, Jones EA, Churchman SM, Baboolal TG, Cuthbert RJ, El-Jawhari JJ, Badawy AM, Alase AA, El-Sherbiny YM, McGonagle D (2017). Interleukin-22 drives the proliferation, migration and osteogenic differentiation of mesenchymal stem cells: a novel cytokine that could contribute to new bone formation in spondyloarthropathies. Rheumatology..

[33] van der Heijde D, Landewe R, Einstein S, Ory P, Vosse D, Ni L, Lin SL, Tsuji W, Davis JC (2008). Radiographic progression of ankylosing spondylitis after up to two years of treatment with etanercept. Arthritis Rheum.

[34] van der Heijde D, Landewe R, Baraliakos X, Houben H, van Tubergen A, Williamson P, Xu W, Baker D, Goldstein N, Braun J (2008). Radiographic findings following two years of infliximab therapy in patients with ankylosing spondylitis. Arthritis Rheum.

[35] Haroon N, Inman RD, Learch TJ, Weisman MH, Lee M, Rahbar MH, Ward MM, Reveille JD, Gensler LS (2013). The impact of tumor necrosis factor alpha inhibitors on radiographic progression in ankylosing spondylitis. Arthritis Rheum.

[36] Maas F, Arends S, Wink FR, Bos R, Bootsma H, Brouwer E, Spoorenberg A (2017). Ankylosing spondylitis patients at risk of poor radiographic outcome show diminishing spinal radiographic progression during long-term treatment with TNF-alpha inhibitors. PloS one.

[37] Baraliakos X, Haibel H, Listing J, Sieper J, Braun J (2014). Continuous long-term anti-TNF therapy does not lead to an increase in the rate of new bone formation over 8 years in patients with ankylosing spondylitis. Ann Rheum Dis.

[38] Molnar C, Scherer A, Baraliakos X, de Hooge M, Micheroli R, Exer P, Kissling RO, Tamborrini G, Wildi LM, Nissen MJ (2018). TNF blockers inhibit spinal radiographic progression in ankylosing spondylitis by reducing disease activity: results from the Swiss Clinical Quality Management cohort. Ann Rheum Dis.

[39] Ritchlin CT, Haas-Smith SA, Li P, Hicks DG, Schwarz EM (2003). Mechanisms of TNF-alpha- and RANKL-mediated osteoclastogenesis and bone resorption in psoriatic arthritis. J Clin Invest.

[40] Kane D, Stafford L, Bresnihan B, FitzGerald O (2003). A prospective, clinical and radiological study of early psoriatic arthritis: an early synovitis clinic experience. Rheumatology..

[41] Eder L, Thavaneswaran A, Chandran V, Gladman DD (2014). Tumour necrosis factor α blockers are more effective than methotrexate in the inhibition of radiographic joint damage progression among patients with psoriatic arthritis. Ann Rheum Dis.

[42] Mease PJ, Gladman DD, Collier DH, Ritchlin CT, Helliwell PS, Liu L, Kricorian G, Chung JB. Etanercept and methotrexate as monotherapy or in combination for psoriatic arthritis: primary results from a randomized, controlled phase 3 trial. Arthritis Rheumatol. 2019;71(7):1112–24. 10.1002/art.40851 PMC6618246 30747501

[43] Nishiyama KK, Pauchard Y, Nikkel LE, Iyer S, Zhang C, DJ MM, Cohen D, Boyd SK, Shane E, Nickolas TL (2015). Longitudinal HR-pQCT and image registration detects endocortical bone loss in kidney transplantation patients. J Bone Miner Res.

[44] de Jong JJA, Christen P, Plett RM, Chapurlat R, Geusens PP, van den Bergh JPW, Muller R, van Rietbergen B (2017). Feasibility of rigid 3D image registration of high-resolution peripheral quantitative computed tomography images of healing distal radius fractures. PloS one.

