# Supplementary material for: Progressive structural bone changes and their relationship with treatment in patients with psoriatic arthritis: a longitudinal HR-pQCT study
Source: Arthritis Res Ther. 2019 Dec 4;21:265. doi: 10.1186/s13075-019-2043-3 (PMC6894233; doi:10.1186/s13075-019-2043-3)
Supplement: Supplementary file 1 — Additional file 1 : Figure S1. Representative image of baseline indexed image registration and slice matching. Figure S2. Examples of bone erosion and enthesiophyte in psoriatic arthritis (PsA) patients at baseline and 5-year follow-up. Figure S3. Baseline volume and dynamics of bone erosion and enthesiophyte. Figure S4. Mean within-subject volume changes in bone erosion and enthesiophyte between patients who achieved and did not achieve sustained DAPSA-LDA. Figure S5. Mean within-subject volume changes in bone erosion and enthesiophyte between patients with and without TNFi throughout 5-year. Table S1. Generalized mixed linear model with change in bone erosion and enthesiophyte volume as dependent variable. [file 13075_2019_2043_MOESM1_ESM.docx]

**Supplementary data for “Progressive structural bone changes and their relationship with treatment: a longitudinal HR-pQCT study in psoriatic arthritis”**

**Authors**

Dongze Wu, PhD^1^, James F Griffith, MD^2^, Steven H.M. Lam, PhD^1^, Priscilla C.H. Wong, FHKCP^1^, Lin Shi, PhD^3^, Edmund K. Li, MD^1^, Isaac T. Cheng, PhD^1^, Tena K. Li, BN^1^, Vivian W. Hung, MPhil^4^, Ling Qin, PhD^4^, Lai-Shan Tam, MD^1^

**Institution**

1. Department of Medicine & Therapeutics, The Prince of Wales Hospital, The Chinese University of Hong Kong, Hong Kong, China

2. Department of Imaging and Interventional Radiology, The Prince of Wales Hospital, The Chinese University of Hong Kong, Hong Kong, China

3. Research Centre for Medical Image Computing, Department of Imaging and Interventional Radiology, The Prince of Wales Hospital, The Chinese University of Hong Kong, Hong Kong, China

4. Bone Quality and Health Centre, Department of Orthopaedics and Traumatology, The Chinese University of Hong Kong, China

**Table of content**

**Figure S1.** Representative image of baseline indexed image registration and slice matching

**Figure S2**. Examples of bone erosion and enthesiophyte in psoriatic arthritis (PsA) patients at baseline and 5-year follow-up.

**Figure S3**. Baseline volume and dynamics of bone erosion and enthesiophyte

**Figure S4**. Mean within-subject volume changes in bone erosion and enthesiophyte between patients who achieved and did not achieve sustained DAPSA-LDA.

**Figure S5**. Mean within-subject volume changes in bone erosion and enthesiophyte between patients with and without TNFi throughout 5-year.

**Table S1**. Generalized mixed linear model with change in bone erosion and enthesiophyte volume as dependent variable


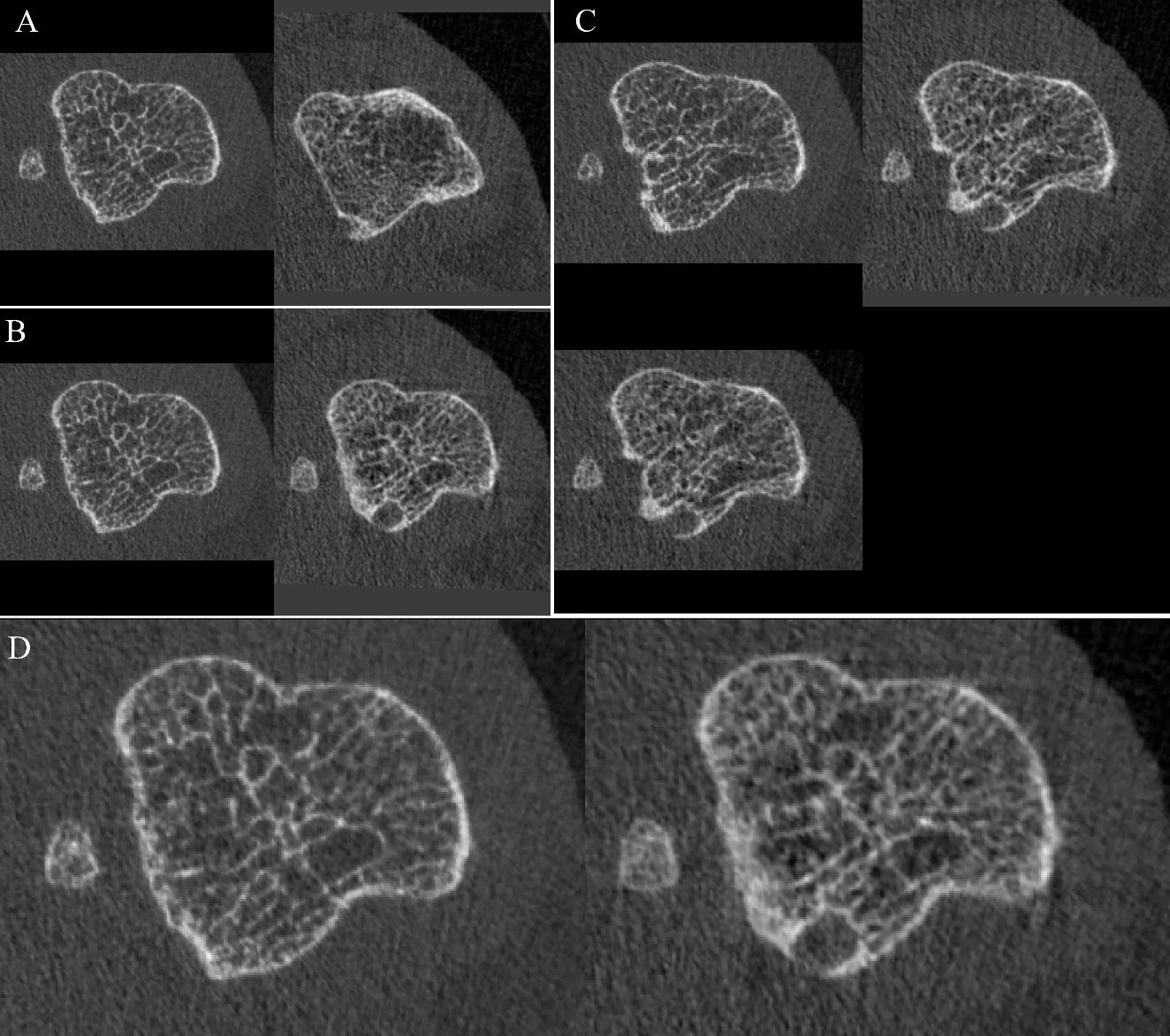


**Figure S1. Representative image of baseline indexed image registration and slice matching**

(A): Crop of metacarpal bone: Crop optimal and wider range VOI for baseline and follow-up HR-pQCT scans, respectively; (B): Baseline indexed image registration: Register the follow-up VOI onto the baseline VOI; (C): Baseline indexed slice matching: Re-slice the follow-up image into the same space of the baseline image; (D): Original baseline and new registered follow-up VOI: It was used to measure volume of bone erosion and enthesiophyte.

**
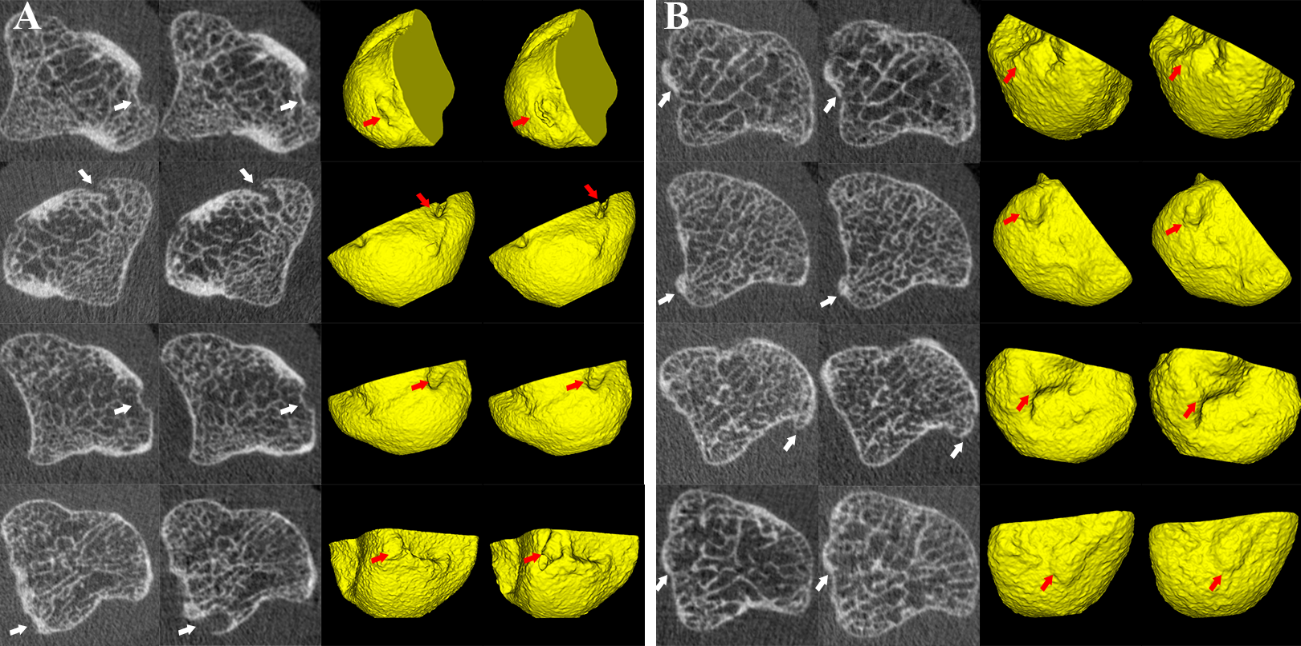
**

**Figure S2**. Examples of bone erosion and enthesiophyte in psoriatic arthritis (PsA) patients at baseline and 5-year follow-up. 2-D and 3-D bone erosion at the metacarpal heads of PsA patients at baseline and 5-year follow-up (A); 2D and 3-D entheisophyte at the metacarpal heads of PsA patients at baseline and 5-year follow-up (B).


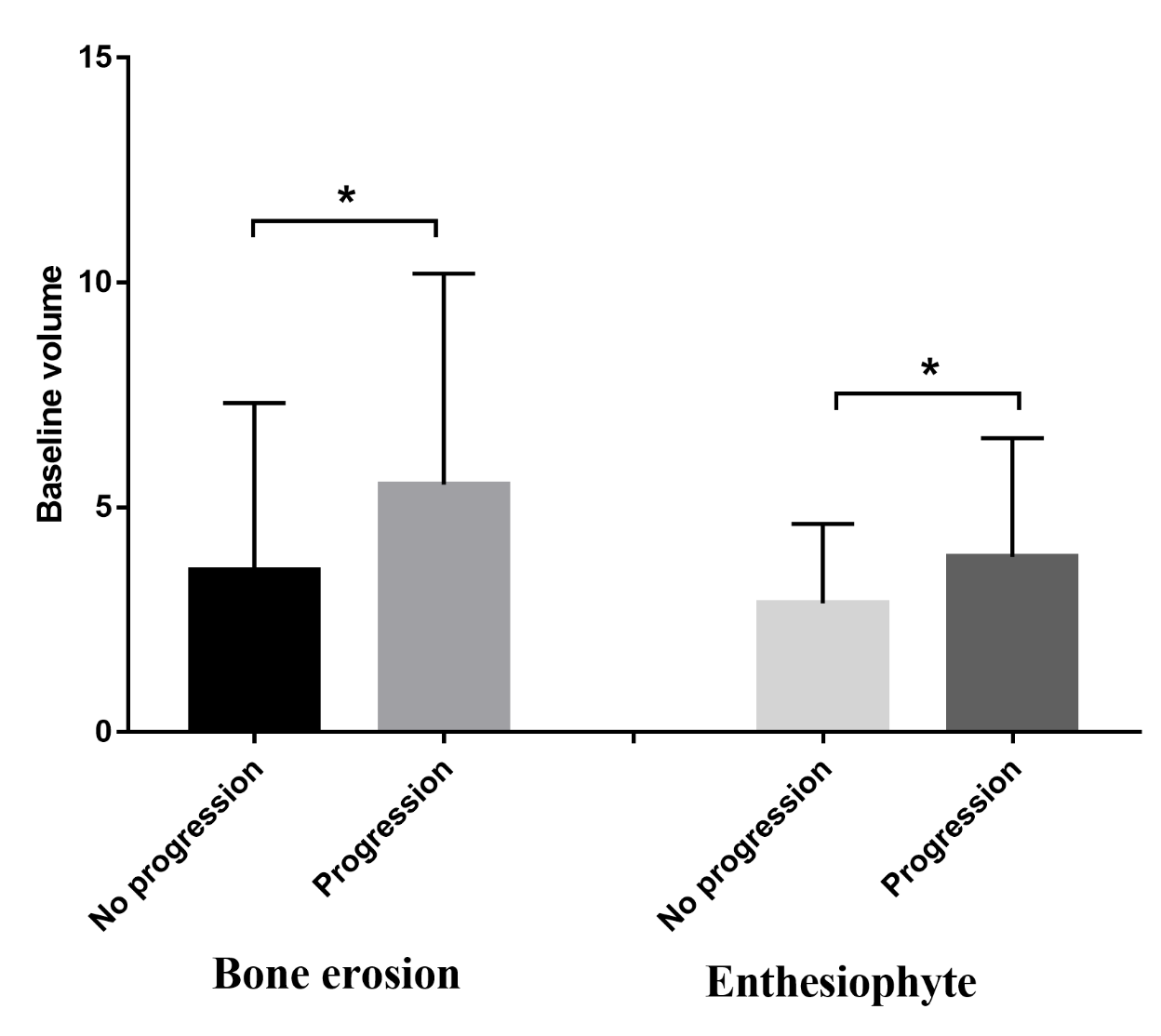


**Figure S3.** Baseline volume and dynamics of bone erosion and enthesiophyte

Baseline volume of bone erosion and enthesiophyte showing non-progression or progression at follow-up.


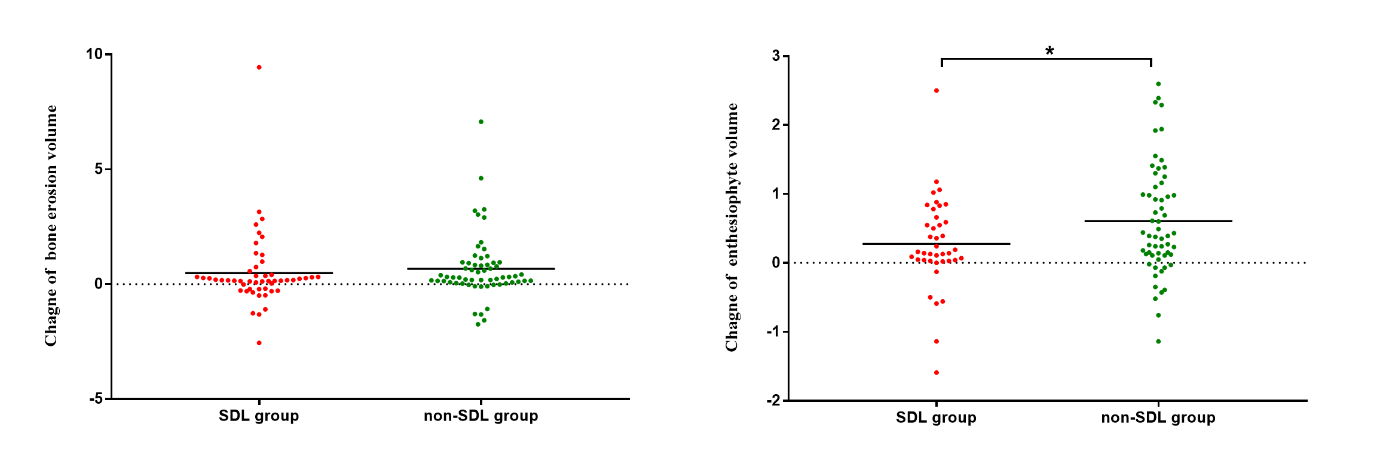


**Figure S4.** **Mean within-subject volume changes in bone erosion and enthesiophyte between patients who achieved and did not achieve sustained DAPSA-LDA.**

Volume change is calculated as follow-up minus baseline expressed as absolute change. A: Change of bone erosion volume; B: Change of enthesiophyte volume. DAPSA: Disease Activity in PSoriatic Arthritis; LDA: low disease activity; SDL group: achieved sustained DAPSA-LDA; non-SDL group: not achieved sustained DAPSA-LDA.


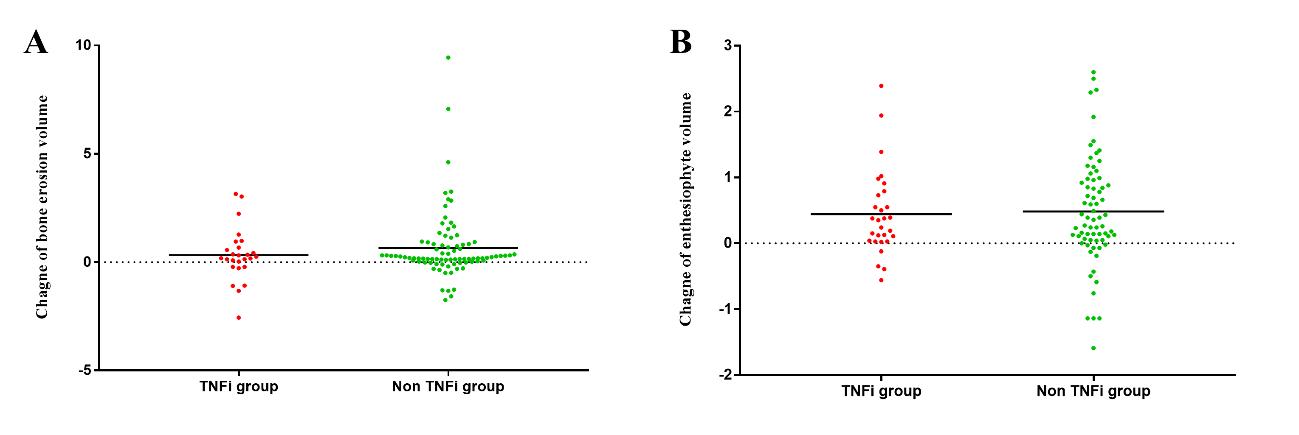


**Figure S5**. Mean within-subject volume changes in bone erosion and enthesiophyte between patients with and without TNFi throughout 5-year. Volume change is calculated as follow-up minus baseline expressed as absolute change. A: Change of bone erosion volume; B: Change of enthesiophyte volume. TNFi group: patients receiving TNFi through 5 years; Non TNFi group: patients not receiving TNFi through 5 years; TNFi: Tumor necrosis factor inhibitor.

**Table S1.** Generalized mixed linear model with change in bone erosion and enthesiophyte volume as dependent variable

|  | Change in bone erosion volume | | Change in enthesiophyte volume | |
| --- | --- | --- | --- | --- |
|  | Regression coefficient | P-value | Regression coefficient | P-value |
| Intercept | -3.215 | 0.270 | 0.285 | 0.813 |
| Male | 0.853 | 0.210 | 0.011 | 0.968 |
| Age | 0.034 | 0.334 | 0.001 | 0.935 |
| BMI | 0.069 | 0.339 | 0.004 | 0.897 |
| Duration of psoriasis | 0.022 | 0.502 | 0.014 | 0.286 |
| Duration of PsA | -0.065 | 0.179 | 0.001 | 0.990 |
| Baseline DAPSA | -0.020 | 0.572 | -0.029 | 0.054 |
| 5-year NSAIDs | -0.378 | 0.551 | 0.213 | 0.420 |
| 5 years of TNFi treatment | -0.674 | 0.406 | -0.072 | 0.823 |
| Sustained DAPSA-LDA | -0.689 | 0.361 | -0.660 | **0.039** |

Significant results are highlighted in bold. BMI: Body Mass Index; DAPSA: Disease Activity in PSoriatic Arthritis; DAPSA-LDA: Disease Activity in PSoriatic Arthritis -Low Disease Activity; TNFi: Tumor Necrosis Factor inhibitor.
